# Screen-Printed Electrodes as Low-Cost Sensors for Breast Cancer Biomarker Detection

**DOI:** 10.3390/s24175679

**Published:** 2024-08-31

**Authors:** Yin Shen, Zhuang Sun, Shichao Zhao, Fei Chen, Peizheng Shi, Ningbin Zhao, Kaiqiang Sun, Chen Ye, Chengte Lin, Li Fu

**Affiliations:** 1 College of Materials and Environmental Engineering, Hangzhou Dianzi University, Hangzhou 310018, China; shenyin@hdu.edu.cn (Y.S.); zhaoshichao@hdu.edu.cn (S.Z.); feichen@hdu.edu.cn (F.C.); 2 Qianwan Institute, Ningbo Institute of Materials Technology and Engineering (NIMTE), Chinese Academy of Sciences, Ningbo 315201, China; sunzhuang@nimte.ac.cn (Z.S.); shipeizheng@nimte.ac.cn (P.S.); zhaoningbin@nimte.ac.cn (N.Z.); sunkaiqiang@nimte.ac.cn (K.S.); yechen@nimte.ac.cn (C.Y.); 3 Key Laboratory of Marine Materials and Related Technologies, Zhejiang Key Laboratory of Marine Materials and Protective Technologies, Ningbo Institute of Materials Technology and Engineering (NIMTE), Chinese Academy of Sciences, Ningbo 315201, China; 4 University of Chinese Academy of Sciences, 19 A Yuquan Rd., Shijingshan District, Beijing 100049, China

**Keywords:** electrochemical sensors, nanomaterials, immunoassays, point-of-care diagnostics, microfluidics

## Abstract

This review explores the emerging role of screen-printed electrodes (SPEs) in the detection of breast cancer biomarkers. We discuss the fundamental principles and fabrication techniques of SPEs, highlighting their adaptability and cost-effectiveness. The review examines various modification strategies, including nanomaterial incorporation, polymer coatings, and biomolecule immobilization, which enhance sensor performance. We analyze the application of SPEs in detecting protein, genetic, and metabolite biomarkers associated with breast cancer, presenting recent advancements and innovative approaches. The integration of SPEs with microfluidic systems and their potential in wearable devices for continuous monitoring are explored. While emphasizing the promising aspects of SPE-based biosensors, we also address current challenges in sensitivity, specificity, and real-world applicability. The review concludes by discussing future perspectives, including the potential for early screening and therapy monitoring, and the steps required for clinical implementation. This comprehensive overview aims to stimulate further research and development in SPE-based biosensors for improved breast cancer management.

## 1. Introduction

Breast cancer remains one of the most prevalent and devastating malignancies affecting women worldwide. As the leading cause of cancer-related deaths among females, it poses a significant global health challenge [1]. Early detection and accurate monitoring of breast cancer progression are crucial for improving patient outcomes and survival rates [2]. In this context, the identification and measurement of specific biomarkers have emerged as powerful tools in breast cancer management, offering the potential for earlier diagnosis, more precise prognosis, and personalized treatment strategies. Biomarkers, which are measurable indicators of biological processes or conditions, play a pivotal role in breast cancer research and clinical practice. These molecular signatures can be proteins, genetic material, or metabolites that are differentially expressed in cancerous tissues or released into bodily fluids. The detection and quantification of breast cancer biomarkers provide valuable insights into the presence, stage, and characteristics of the disease [3,4]. Common breast cancer biomarkers include proteins such as HER2 (Human Epidermal Growth Factor Receptor 2), CA15-3 (Cancer Antigen 15-3), and CEA (Carcinoembryonic Antigen), as well as genetic markers like BRCA1 and BRCA2 mutations and specific microRNAs. These biomarkers can be proteins, genetic materials, or metabolites that are differentially expressed in cancerous tissues or released into bodily fluids. Table 1 summarizes some of the most common breast cancer biomarkers, their molecular sizes, utility in breast cancer management, and typical serum levels.

**Figure 1:**
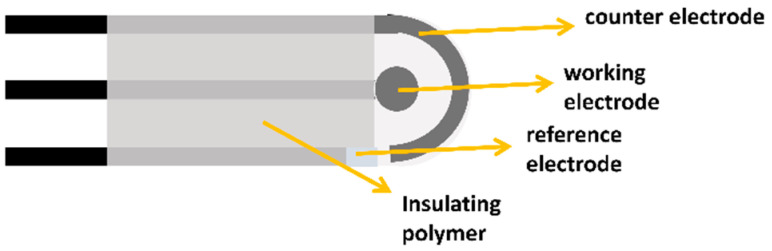
Scheme of SPE.

Despite the immense potential of biomarkers in breast cancer management, current detection methods face several challenges. Traditional techniques such as enzyme-linked immunosorbent assays (ELISA) [12], immunohistochemistry [13], and polymerase chain reaction (PCR) [14] are often time-consuming, expensive, and require sophisticated laboratory equipment and trained personnel. These limitations hinder the widespread implementation of biomarker testing, particularly in resource-limited settings or for frequent monitoring during treatment. Moreover, the complexity and variability of breast cancer necessitate the simultaneous detection of multiple biomarkers for comprehensive disease assessment, further complicating the analytical process. In light of these challenges, there is a pressing need for innovative, cost-effective, and accessible technologies for breast cancer biomarker detection. Screen-printed electrodes (SPEs) have emerged as a promising platform to address this need, offering a unique combination of simplicity, affordability, and analytical performance [15]. SPEs are miniaturized electrochemical sensors fabricated through a screen-printing process, which allows for the mass production of reproducible and disposable devices [16]. These electrodes typically consist of a working electrode, a counter electrode, and a reference electrode printed on a solid substrate, creating a complete electrochemical cell in a compact format (Figure 1). The potential of SPEs as low-cost sensors for breast cancer biomarker detection stems from several key advantages. Firstly, their simple and inexpensive fabrication process makes them highly suitable for large-scale production and single-use applications, addressing concerns of cross-contamination and reducing the need for complex cleaning procedures. Secondly, SPEs offer remarkable versatility in terms of electrode materials and surface modifications, allowing for tailored designs to detect specific biomarkers with high sensitivity and selectivity. The ability to incorporate various nanomaterials, polymers, and biomolecules onto the electrode surface further enhances their analytical capabilities [17]. Furthermore, SPEs are compatible with a wide range of electrochemical techniques, including amperometry, voltammetry, and impedance spectroscopy, providing multiple avenues for biomarker detection and quantification [18]. Their miniaturized format requires only small sample volumes, which is particularly advantageous when working with limited biological specimens [19]. Additionally, the portability and ease of use of SPE-based sensors make them well-suited for point-of-care (POC) applications, potentially enabling rapid and on-site breast cancer biomarker testing [20]. In terms of specificity, SPE-based biosensors can be tailored for selective detection of breast cancer biomarkers through careful selection of biorecognition elements and surface modification strategies. For instance, the use of aptamers or molecularly imprinted polymers on SPEs can provide highly specific binding sites for target molecules, reducing non-specific interactions and improving selectivity compared to traditional immunoassay platforms. When comparing SPE-based biosensors to traditional sensing methods in real-world environments, several advantages become apparent. The disposable nature of SPEs eliminates the need for complex cleaning procedures and reduces the risk of cross-contamination, which is particularly beneficial in point-of-care settings. Furthermore, the miniaturized design of SPEs requires smaller sample volumes compared to conventional methods like ELISA, making them more suitable for analyzing limited biological specimens.

Recent advancements in SPE technology have significantly improved their performance in biosensing applications. The development of novel electrode materials, such as carbon nanotubes, graphene, and metal nanoparticles, has enhanced the sensitivity and electron transfer properties of SPEs [21]. Innovative surface modification strategies have enabled the immobilization of various biorecognition elements, including antibodies, aptamers, and molecularly imprinted polymers, expanding the range of detectable biomarkers and improving specificity [22]. Moreover, the integration of SPEs with microfluidic systems and multiplexed designs has opened up new possibilities for the simultaneous detection of multiple breast cancer biomarkers, providing a more comprehensive disease profile [23]. The application of SPEs in breast cancer biomarker detection spans a wide spectrum of molecular targets. Protein biomarkers, such as HER2 [24], CA15-3 [25], and CEA [26], have been successfully detected using SPE-based immunosensors, often achieving sensitivities comparable to or exceeding those of conventional immunoassays. Genetic biomarkers, including BRCA1/2 mutations and circulating microRNAs [27,28], have been analyzed using SPE-based DNA sensors and aptasensors, offering rapid and sensitive alternatives to PCR-based methods.

Despite the significant progress in SPE-based breast cancer biomarker detection, several challenges remain to be addressed for their widespread clinical adoption. These include further improvements in sensitivity and specificity, especially in complex biological matrices, as well as enhancing the long-term stability and reproducibility of the sensors. The development of standardized fabrication processes and quality control measures is crucial for ensuring consistent performance across different batches of SPEs. Furthermore, the integration of SPE-based sensors with user-friendly readout devices and data analysis systems is essential for their practical implementation in clinical settings. This review aims to explore the potential of SPEs as low-cost, sensitive, and versatile platforms for breast cancer biomarker detection. We provide a comprehensive overview of SPE technology, from fundamental principles and fabrication techniques to advanced modification strategies that enhance biosensor performance. The review delves into the application of SPEs for detecting various types of breast cancer biomarkers, including proteins, genetic markers, and metabolites, highlighting innovative approaches and recent advancements in the field. We also discuss the progress towards multiplexed detection of biomarker panels, which offer a more comprehensive assessment of breast cancer status. Furthermore, this review examines the current challenges facing SPE-based breast cancer biosensors and explores future perspectives, including emerging materials, integration with microfluidics and wearable devices, and considerations for clinical implementation. By synthesizing the latest developments and identifying future research directions, this review aims to stimulate further advancements in SPE-based biosensors for breast cancer management, potentially leading to more accessible and effective tools for early detection and personalized treatment strategies.

## 2. Screen-Printed Electrodes: Fundamentals and Fabrication

SPEs have emerged as a cornerstone technology in the field of electrochemical biosensing, offering a unique combination of simplicity, cost-effectiveness, and analytical performance. At their core, SPEs are miniaturized electrochemical cells fabricated through a process akin to traditional screen-printing techniques used in the textile industry. This innovative approach to electrode production has revolutionized the development of portable and disposable sensing platforms, particularly in the realm of biomedical diagnostics. The basic structure of an SPE typically comprises three essential components: a working electrode, a counter electrode, and a reference electrode (Figure 1). These elements are printed in close proximity on a solid substrate, creating a complete electrochemical cell within a compact footprint [22,29,30]. The working electrode serves as the primary sensing surface where the electrochemical reactions of interest occur. It is often composed of conductive materials such as carbon, gold, or platinum [31], chosen based on the specific requirements of the target analyte and detection method. The counter electrode, usually made of the same material as the working electrode, completes the electrical circuit and allows current to flow through the cell. The reference electrode, commonly silver/silver chloride (Ag/AgCl), provides a stable potential against which the working electrode potential is measured, ensuring accurate and reproducible measurements.

**Figure 2:**
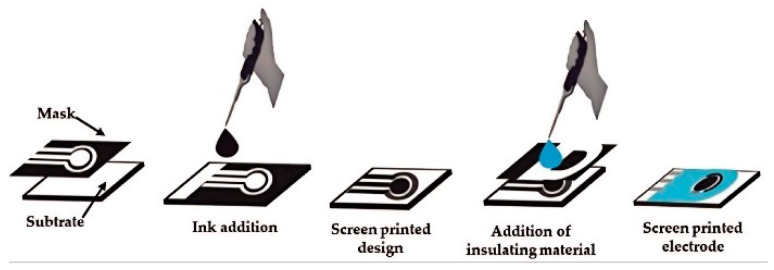
Schematic representation of the manufacturing process of SPEs. Reprinted with permission from Ref. [16]. Copyright 2021 Elsevier.

The manufacturing process of SPEs involves several key steps that contribute to their unique properties and advantages (Figure 2). The process begins with the preparation of conductive inks or pastes, which form the basis of the electrode materials. These inks are carefully formulated to achieve the desired conductivity, viscosity, and adhesion properties [32]. Carbon-based inks, often incorporating graphite or carbon black, are widely used due to their excellent conductivity, wide potential window, and low background currents [33]. Metallic inks, such as those containing gold or silver particles, offer enhanced conductivity and are particularly useful for certain biosensing applications [34]. Once the inks are prepared, the actual printing process commences. A specially designed screen, typically made of a fine mesh stretched over a frame, is used to define the electrode pattern. The screen contains open areas corresponding to the desired electrode design, while the rest of the mesh is blocked with a non-permeable material [35]. The conductive ink is then forced through the open areas of the screen onto the substrate using a squeegee, depositing a precise pattern of electrode material. This process is repeated for each electrode component, with careful alignment ensuring the correct spatial arrangement of the working, counter, and reference electrodes. The choice of substrate material plays a crucial role in the overall performance and applicability of SPEs. Common substrate materials include various polymers such as polyethylene terephthalate (PET) [36], polyvinyl chloride (PVC) [37], and ceramic materials [38]. These substrates are selected based on their mechanical stability, chemical resistance, and compatibility with the intended application environment. The flexibility of substrate choice allows for the production of both rigid and flexible SPEs, expanding their potential use in diverse sensing scenarios. Following the printing process, the electrodes undergo a curing step to solidify the ink and ensure proper adhesion to the substrate. This may involve heat treatment, UV exposure, or simply air drying, depending on the specific ink formulation. In some cases, additional layers may be printed on top of the electrodes, such as insulating materials to define the active electrode area or protective coatings to enhance durability.

The advantages of SPEs for biosensing applications are manifold. Their miniaturized design requires only small sample volumes, which is particularly beneficial when working with limited biological specimens. The disposable nature of SPEs eliminates concerns of cross-contamination between samples and negates the need for time-consuming cleaning procedures. Moreover, the mass production capability of screen-printing technology translates to low manufacturing costs, making SPEs an economically viable option for widespread use in both research and clinical settings. Another significant advantage of SPEs lies in their versatility and ease of modification. The electrode surface can be readily functionalized with a wide range of biomolecules, nanomaterials, and polymers to enhance sensitivity, selectivity, and overall analytical performance. This adaptability allows for the development of tailored sensing platforms for specific biomarkers or classes of analytes. Furthermore, the planar design of SPEs facilitates their integration into portable, handheld devices, paving the way for POC diagnostic applications.

Recent advances in SPE fabrication techniques have further expanded their capabilities and potential applications. One notable development is the incorporation of nanomaterials directly into the electrode inks. Carbon nanotubes (CNTs), graphene, and metal nanoparticles have been successfully integrated into SPE formulations [39], resulting in enhanced conductivity, increased surface area, and improved electrocatalytic properties. These nanocomposite electrodes offer superior sensitivity and lower detection limits compared to traditional carbon-based SPEs. Advancements in printing technology have also contributed to the evolution of SPE fabrication. High-resolution printing techniques, such as inkjet printing and microcontact printing, have enabled the production of SPEs with finer features and more precise control over electrode geometry. This has led to the development of microelectrode arrays [40,41] and interdigitated electrode designs [42,43], which offer improved mass transport characteristics and enhanced sensitivity for certain applications. The integration of SPEs with microfluidic systems represents another frontier in their development [23,44]. By combining the electrochemical sensing capabilities of SPEs with the precise fluid handling and sample processing capabilities of microfluidic devices, researchers have created powerful lab-on-a-chip platforms for complex bioanalytical applications. These integrated systems offer the potential for automated, multi-step analyses with minimal sample and reagent consumption.

## 3. Modification Strategies for SPEs in Breast Cancer Biomarker Detection

The evolution of SPEs in breast cancer biomarker detection has been greatly enhanced by the development of various modification strategies. These approaches aim to improve the sensitivity, selectivity, and overall performance of SPEs, making them more suitable for the complex task of detecting cancer-specific molecules. Among the most prominent modification strategies are those involving nanomaterials, polymers, biomolecule immobilization techniques, and methods to achieve multiplexing capabilities.

### 3.1. Nanomaterial-Based Screen-Printing Pastes

**Figure 3:**
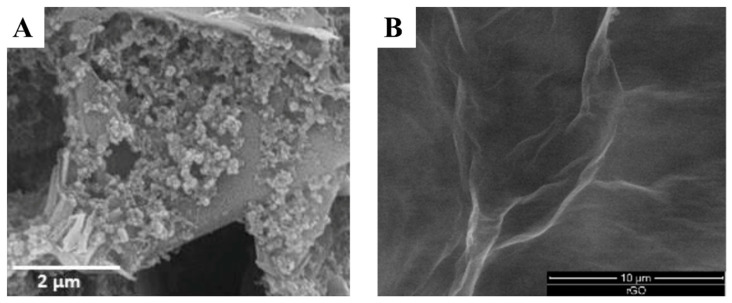
SEM images of (**A**) MWCNTs and (**B**) rGO modified SPEs. Reprinted with permission from Refs. [45,46]. Copyright 2016 MDPI and 2016 Elsevier.

Nanomaterial-based modifications have revolutionized the field of SPE-based biosensors for breast cancer detection. Carbon nanomaterials, such as CNTs and graphene, have gained significant attention due to their exceptional electrical properties and high surface-to-volume ratios. When incorporated into SPEs, these materials dramatically increase the electrode’s active surface area, enhancing electron transfer rates and improving sensitivity towards breast cancer biomarkers. Multi-walled carbon nanotubes (MWCNTs), for instance, have been used to modify SPEs for the detection of HER2 [45]. The three-dimensional network formed by MWCNTs not only increases the electrode’s conductivity but also provides numerous binding sites for biorecognition elements, resulting in improved detection limits. Graphene and its derivatives, such as reduced graphene oxide (rGO), have also shown great promise in SPE modification for breast cancer biomarker detection. The two-dimensional structure of graphene offers an expansive surface for biomolecule immobilization while maintaining excellent electrical conductivity. SPEs modified with graphene-based materials have demonstrated enhanced sensitivity towards various breast cancer markers, including CEA [46,47] and CA15-3 [25]. The unique properties of graphene also allow for easier functionalization, enabling the attachment of specific biorecognition elements for targeted biomarker detection. Figure 3 shows the morphology of MWCNT and rGO-modified SPEs.

Metal nanoparticles represent another class of nanomaterials extensively used in SPE modification for breast cancer diagnostics. AuNPs, in particular, have been widely employed due to their biocompatibility, stability, and ability to facilitate electron transfer [48]. When incorporated into SPEs, AuNPs can serve as excellent platforms for the immobilization of antibodies or aptamers specific to breast cancer biomarkers. The large surface area of AuNPs allows for a high density of biorecognition elements, leading to improved sensitivity and lower detection limits. AgNPs have also found in some applications in SPE modification for breast cancer biomarker detection [49]. These metallic nanoparticles often exhibit catalytic properties that can enhance the electrochemical response of specific biomarkers.

**Figure 4:**
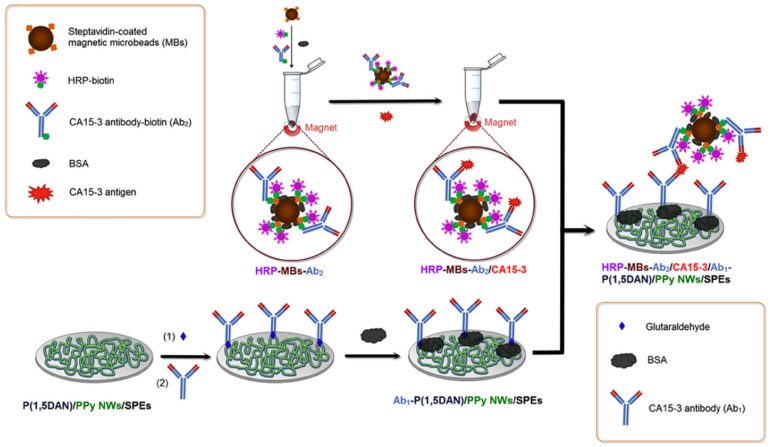
Scheme of an immunosensor based on P(1,5DAN)/PPy NWs bilayer and magnetic beads for CA15-3 detection. Reprinted with permission from Ref. [50]. Copyright 2017 Elsevier.

Polymer-based modifications offer another versatile approach to enhancing SPE performance in breast cancer biomarker detection. Conductive polymers, such as polypyrrole (PPy) and poly(1,5-diaminonaphthalene) [P(1,5DAN)], can be electropolymerized directly onto SPE surfaces, creating a three-dimensional network that increases the electrode’s surface area and improves its electrical properties. For example, Nguyen et al. [50] developed an electrochemical immunosensor for detecting the CA 15-3 using a bilayer film of PPy nanowires (PPy NWs) and P(1,5DAN) on SPE. The PPy NWs inner layer provided high surface area and conductivity, while the P(1,5DAN) outer layer enabled antibody immobilization via amino groups. Magnetic beads conjugated with secondary antibodies and horseradish peroxidase were used for signal amplification (Figure 4). The optimized immunosensor exhibited a linear range of 0.05–20 U/mL and a detection limit of 0.02 U/mL for CA 15-3. The PPy NWs/P(1,5DAN) bilayer showed superior performance compared to single-layer films, with about 2 times higher current response than PPy NWs alone and 4.5 times higher than P(1,5DAN) alone. The approach combined the advantages of both polymers—PPy NWs provided high surface area and conductivity while P(1,5DAN) enabled covalent antibody attachment. This bilayer strategy demonstrated the potential for developing sensitive electrochemical immunosensors with enhanced biomolecule immobilization and electron transfer.

**Figure 5:**
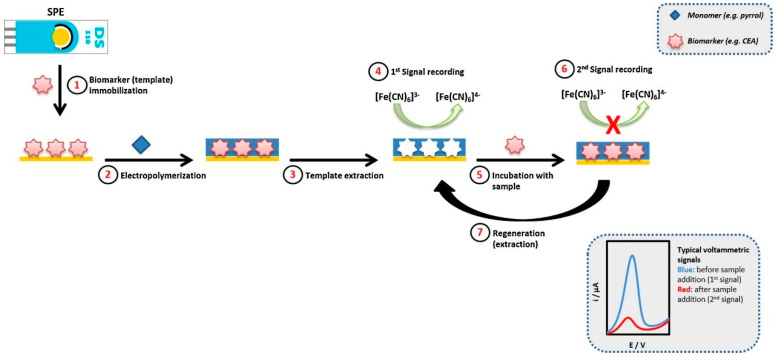
Scheme of construction and operation of the MIP/Au-SPE. More details can be found in the text. Reprinted with permission from Ref. [51]. Copyright 2018 Elsevier.

Molecularly imprinted polymers (MIPs) represent a unique class of synthetic materials that can be tailored to recognize specific breast cancer biomarkers. When used to modify SPEs, MIPs can create highly selective binding sites that mimic the recognition properties of natural antibodies. This approach has been successfully applied to the detection of various breast cancer-associated proteins and small molecules, offering a robust and cost-effective alternative to traditional antibody-based assays. For example, Pacheco et al. [51] developed a novel electrochemical sensor for detecting the CA 15-3 using a MIP on a screen-printed gold electrode (Au-SPE). The MIP was created through direct surface imprinting of CA 15-3 on the Au-SPE, followed by electropolymerization of 2-aminophenol. The resulting MIP/Au-SPE sensor demonstrated high selectivity and sensitivity for CA 15-3 detection (Figure 5). Voltammetric analysis using hexacyanoferrate(II/III) as a redox probe revealed a linear relationship between the peak current intensity and the logarithm of CA 15-3 concentration in the range of 5–50 U/mL. The sensor achieved a detection limit of 1.5 U/mL, which was well below the clinical cut-off value of 25 U/mL. Selectivity studies showed some interference from HER2-ECD but minimal interference from cystatin C. When applied to spiked human serum samples, the sensor exhibited recoveries of 72–87% with relative standard deviations of 5–9%.

Hydrogel-based modifications have also gained attention in SPE development for breast cancer diagnostics. These water-swollen polymeric networks can provide a three-dimensional environment that mimics physiological conditions, potentially improving the stability and activity of immobilized biomolecules. For example, Chocholova et al. [52] developed an advanced impedimetric biosensor using a novel approach combining zwitterionic hydrogel-modified interfaces and glycoprofiling of HER2 protein. The hydrogel significantly reduced non-specific protein adsorption, enhancing the sensor’s performance in complex biological samples. Anti-HER2 antibodies were covalently attached to the hydrogel surface for specific HER2 detection. The biosensor achieved a remarkable detection limit of 5 pg/mL (77 fM) for HER2. Notably, the CBAmN3-1 hydrogel-based sensor demonstrated superior specificity with only 4.4% non-specific interactions compared to 6.2% for the CBAmN3-2 variant. The researchers applied this ultrasensitive biosensor to glycoprofile HER2 in human serum samples using lectins, successfully distinguishing between a high-risk individual without breast cancer and a patient with stage 2 breast cancer.

### 3.2. Immobilization Strategies

**Figure 6:**
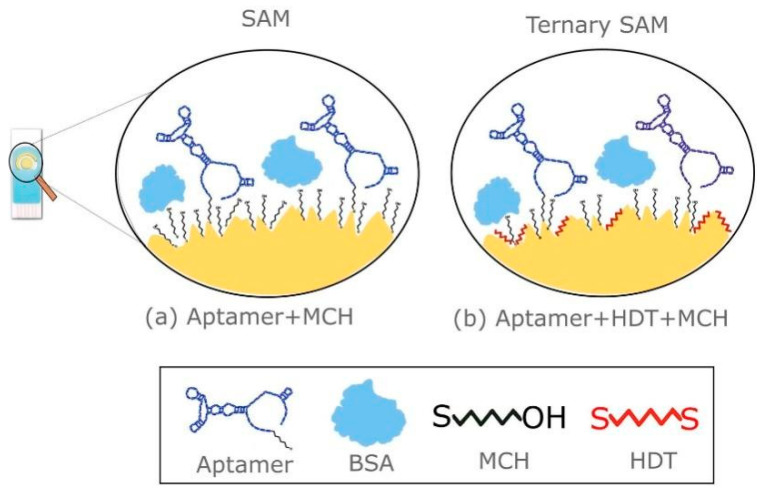
Surface architecture for SAM and ternary SAM. In (**a**) SPE modified with DNA Aptamer and MCH to form a SAM. In (**b**) SPE modified with DNA Aptamer, MCH and HDT to form a ternary SAM. The HDT can adopt a vertical or horizontal configuration, blocking the remaining spaces on the irregular surface. Reprinted with permission from Ref. [54]. Copyright 2021 Elsevier.

Biomolecule immobilization techniques play a crucial role in the development of effective SPE-based biosensors for breast cancer detection. The choice of immobilization method can significantly impact the sensor’s sensitivity, stability, and overall performance. Covalent attachment of biomolecules to the electrode surface is often preferred due to its stability and reproducibility. Common approaches include the use of cross-linking agents such as glutaraldehyde or carbodiimide chemistry to form stable bonds between the biomolecules and the electrode surface [53]. Self-assembled monolayers (SAMs) have also been extensively used for biomolecule immobilization on SPEs. This technique involves the spontaneous formation of organized molecular layers on the electrode surface, providing a well-defined and controllable interface for subsequent attachment of biorecognition elements. For example, Ferreira et al. [54] developed and optimized an electrochemical aptasensor for detecting HER2 protein using Au-SPE. Two sensing platforms were created: a SAM and a ternary SAM. The SAM platform was composed of thiolated DNA aptamers specific for HER2 and 1-mercapto-6-hexanol, while the ternary SAM included 1,6-hexanethiol (Figure 6). In phosphate-buffered saline, the SAM platform demonstrated a sensitivity of 8.0% per decade and a LOD of 108.4 pg/mL. When tested in undiluted human serum, the SAM platform exhibited a sensitivity of 4.1% per decade and an LOD of 179 pg/mL. The study emphasized the use of SAM to create an organized and oriented layer for biosensing applications.

### 3.3. Multiplex Sensors

**Figure 7:**
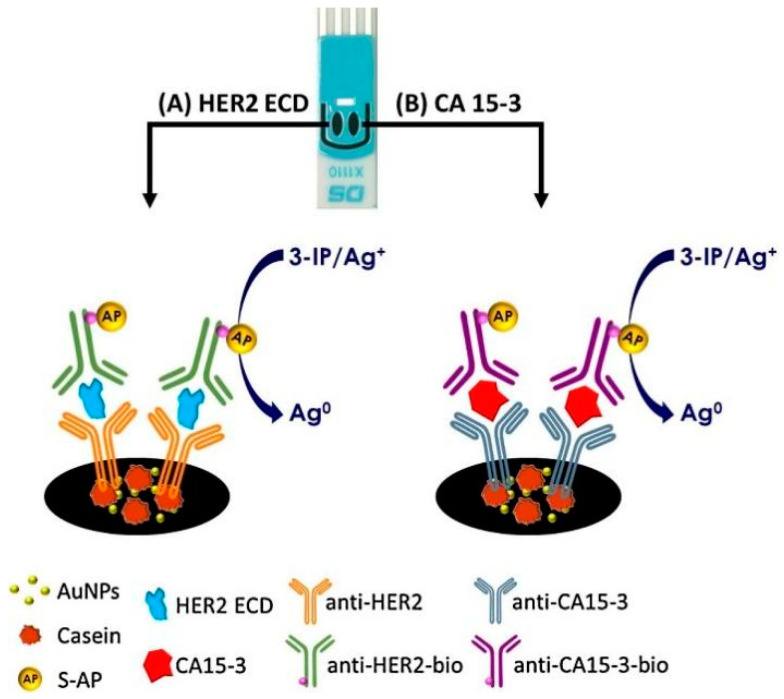
Schematic representation of the immunoassay for HER2 and CA 15-3 on a bi-SPCE-AuNP. More details can be found in the text. Reprinted with permission from Ref. [55]. Copyright 2018 Elsevier.

The development of multiplexing capabilities in SPE-based biosensors represents a significant advancement in breast cancer biomarker detection. Multiplexed assays allow for the simultaneous detection of multiple biomarkers, providing a more comprehensive picture of the disease state and potentially improving diagnostic accuracy. For example, Marques et al. [55] developed the first multiplexed electrochemical immunosensor for simultaneously detecting two breast cancer biomarkers: CA 15-3 and HER2. The researchers optimized a sandwich-type assay on dual SPE modified with AuNPs (Figure 7). They achieved limits of detection of 2.9 ng/mL for HER and 5.0 U/mL for CA 15-3, both below clinical cutoff values. The sensor showed linear responses from 9.8–50 ng/mL for HER2-ECD and 17–70 U/mL for CA 15-3. Precision was adequate with relative standard deviations around 5%.

## 4. SPE-Based Biosensors for Breast Cancer Biomarkers

SPE-based biosensors have emerged as powerful tools for the detection and quantification of various breast cancer biomarkers. These biosensors offer the potential for rapid, sensitive, and cost-effective analysis of a wide range of molecular indicators associated with breast cancer development, progression, and treatment response. The versatility of SPE-based platforms allows for the detection of diverse biomarker types, including proteins, genetic markers, and metabolites, each providing unique insights into the complex biology of breast cancer.

### 4.1. HER2

**Figure 8:**
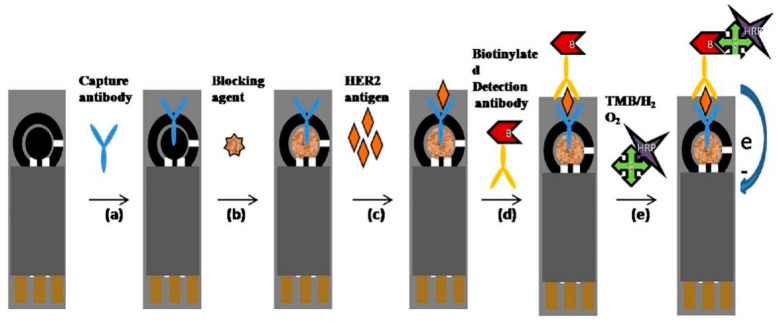
Schematic diagram of immunosensor preparation and electro-catalytic reduction in TMB:H_2_O_2_ substrate by the HRP attached to the immunoassay system. More details can be found in the text. Reprinted with permission from Ref. [57]. Copyright 2017 Elsevier.

Protein biomarkers have long been the cornerstone of breast cancer diagnostics and monitoring, and SPE-based biosensors have shown remarkable capabilities in their detection. HER2 is a prime example of a critical breast cancer protein biomarker that has been successfully targeted using SPE-based approaches. Overexpression of HER2 is associated with aggressive tumor behavior and poor prognosis, making its accurate detection crucial for treatment decisions. SPE-based immunosensors for HER2 typically involve the immobilization of anti-HER2 antibodies on the electrode surface. For example, Al-Khafaji et al. [56] developed a simple and sensitive electrochemical immunoassay for detecting HER2. The assay utilized antibody-functionalized magnetic beads coupled with SPE in a sandwich format. After optimizing various parameters, including antibody concentrations and incubation times, the assay demonstrated a linear response for HER2 in the clinically relevant range of 0–15 ng/mL, with a detection limit of 6 ng/mL. The method showed good reproducibility, with an average coefficient of variation of 7%. When tested on spiked serum samples, the assay successfully discriminated between different HER2 concentrations, even in the complex serum matrix. Analysis of real serum samples from breast cancer patients revealed a correlation between signal intensity and cancer stage, with samples from patients with advanced metastasis showing significantly higher signals (2.5–7.3 μA) compared to those without metastasis or with lymph node involvement (0.15–0.23 μA). Tallapragada et al. [57] developed an immunosensor using SPEs for detecting HER2 antigen in human serum samples. The immunosensor employed an ELISA format without any surface modification of the working electrode (Figure 8). The immunosensor demonstrated a linear response to HER2 concentrations in two ranges: 5–20 ng/mL and 20–200 ng/mL. The LOD and LOQ were 4 ng/mL and 5 ng/mL, respectively. The device showed good reproducibility with a relative standard deviation of 0.7% for six electrodes. When tested on real biological samples, the immunosensor detected an average abnormal serum HER2 level of 34 ng/mL in invasive breast cancer patients, while healthy individuals and non-invasive breast cancer patients showed an average level of 13.45 ng/mL.

One innovative approach for HER2 detection utilizes AuNPs-modified SPEs functionalized with HER2-specific aptamers. This design capitalizes on the high surface area and excellent conductivity of AuNPs, combined with the specificity of aptamer recognition. The binding of HER2 to the aptamers causes measurable changes in the electrode’s electrochemical properties, allowing for quantitative detection. For example, Harahsheh et al. [24] developed an aptamer-based biosensor for detecting HER2. The researchers used a screen-printed carbon electrode modified with AuNPs to immobilize HER2-specific aptamers. The biosensor exhibited a wide linear detection range from 0.001 to 100 ng/mL and achieved a very low detection limit of 0.001 ng/mL. It demonstrated high sensitivity, with a response of 52.85 μA per decade of HER2 concentration. The aptasensor showed a fast binding time of only 5 min and maintained stable performance for 72 h with a relative standard deviation of around 4%. Selectivity studies revealed minimal cross-reactivity with interfering substances, with interference levels not exceeding 10% of the HER2 response. When exposed to a mixture of HER2 and high concentrations of interfering substances, the extent of interference was approximately 8%. These results indicated that the developed aptasensor offered a promising platform for selective and sensitive HER2 detection in breast cancer diagnostics. Table 2 shows the key characteristics of SPE-based electrochemical sensors for the detection of HER2.

### 4.2. CA15-3

**Figure 9:**
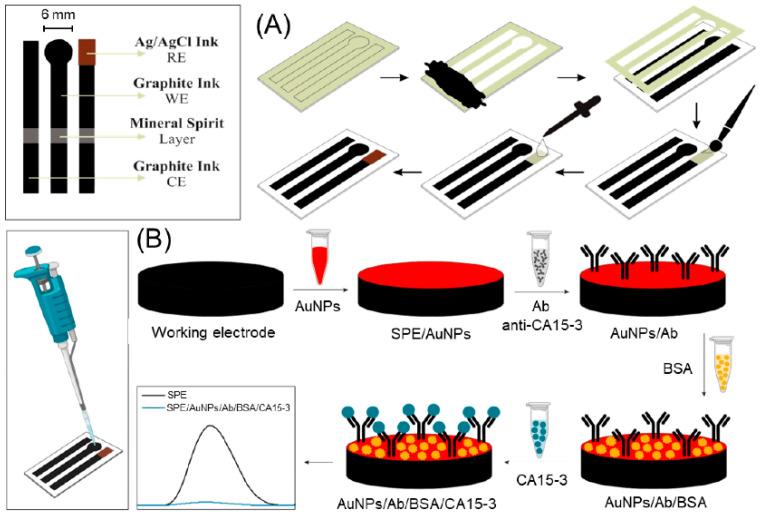
(**A**) Fabrication and (**B**) modification of SPE with AuNPs, anti-CA 15-3, and BSA to develop an immunosensor for CA 15-3 determination. Modification occurred through the addition of modifiers to the electrode surface using a pipette. After incubation with CA 15-3, a reduction in the electrochemical signal was observed, as shown in the voltammogram. Reprinted with permission from Ref. [67]. Copyright 2024 MDPI.

CA15-3 is another crucial protein biomarker for breast cancer that has been extensively studied using SPE-based biosensors. As a mucin-type glycoprotein shed from tumor cells, CA15-3 levels in serum correlate with tumor burden and are valuable for monitoring treatment response and disease recurrence. SPE-based sensors for CA15-3 often employ sandwich-type immunoassay configurations, where the target protein is captured between two specific antibodies. For example, Oliveira et al. [67] developed a disposable voltammetric immunosensor aimed at determining and quantifying the biomarker CA 15-3 in human saliva and serum samples. The sandwich-type immunoassay was employed to enhance specificity and sensitivity, leveraging antigen–antibody interactions alongside a redox species, ferrocyanide potassium, for indirect antigen determination (Figure 9). The immunosensor demonstrated a linear detection range from 2 to 16 U/mL with a sensitivity of 0.012 µA/U mL^−1^. Its LOD was 0.56 U/mL, and the limit of quantification was 1.88 U/mL. The device showed minimal signal interference from other substances, with a response variation of only 4.94%. Reproducibility was confirmed with a relative standard deviation of 5.65%. A notable example of CA15-3 detection involves an MWCNT-modified SPE functionalized with a primary anti-CA15-3 antibody [68]. The study developed an electrochemical immunosensor using a composite of doped poly(2-chloroaniline) (dP2ClAn) and MWCNT to detect the breast cancer biomarker CA15-3 in serums. The composite was coated layer-by-layer onto an SPE, with anti-CA15-3 antibodies successfully immobilized through physical adsorption without a crosslinking agent. The immunosensor demonstrated excellent performance within the range of 5 to 100 U/mL, with a detection limit of 0.66 U/mL and a sensitivity of 254.67 μA/mL/U/cm^2^. Table 3 shows the key characteristics of SPE-based electrochemical sensors for the detection of CA15-3.

### 4.3. CEA

**Figure 10:**
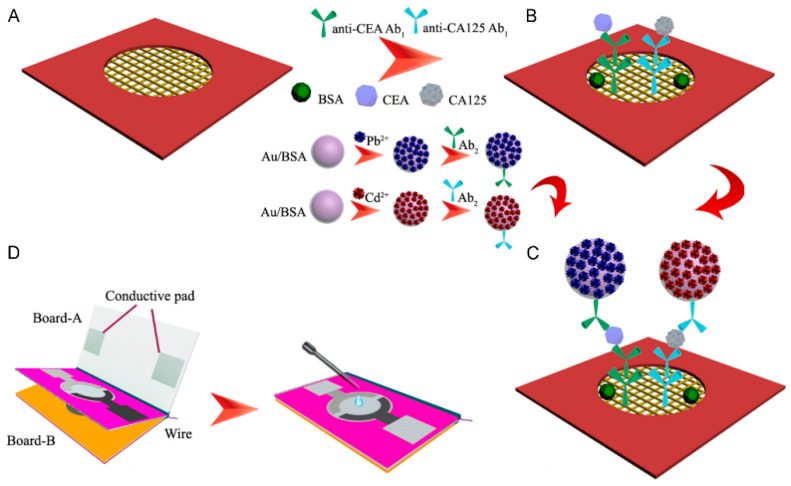
Scheme of the fabrication and assay procedures of 3D origami EC immunodevice. (**A**) AuNRs-PWE: growth of an interconnected AuNRs layer on the surfaces of cellulose fibers in bare PWE. (**B**) After immobilization with Ab_1_s, BSA, CEA and CA125. (**C**) After incubating with the designed tracers. (**D**) After modification, this immunodevice was integrated with a transparent device-holder, the device-holder was clamped closely and 40 µL supporting electrolyte was added for electrochemical assay. Reprinted with permission from Ref. [75]. Copyright 2015 Elsevier.

CEA is a well-established tumor marker that, while not specific to breast cancer, can provide valuable information when monitored alongside other biomarkers. SPE-based biosensors for CEA detection have explored various innovative approaches to enhance sensitivity and specificity. One such approach involves the use of a dual-signal amplification system. Ma et al. [75] developed a novel 3D origami electrochemical immunodevice for sensitive POC testing of CEA using a dual-signal amplification strategy. The device employed Au nanorod-modified SPE (AuNRs-SPE) as the sensor platform and metal ion-coated Au/bovine serum albumin nanospheres as tracing tags to achieve high sensitivity (Figure 10). The dual-signal amplification approach allowed simultaneous detection of CEA and cancer antigen 125 (CA125) with wide linear ranges spanning over 4 orders of magnitude. Detection limits as low as 0.08 pg/mL for CEA and 0.06 mU/mL for CA125 were achieved. The device exhibited good stability, with only a 4% decrease in electrochemical response after 30 days of storage (). Relative standard deviations of 2.67% and 2.82% for CEA and CA125 detection indicated good precision and reproducibility. When tested on human serum samples, the results showed good agreement with commercial electrochemiluminescent single-analyte tests, with relative errors below 3.6%. The combination of AuNRs-SPE and metal ion tracers provided significant signal amplification, enabling highly sensitive multiplex detection of cancer biomarkers in a simple, low-cost device suitable for POC applications. Shi et al. [76] developed a novel smartphone-based electrochemical aptasensing platform for the POC testing of CEA. The platform utilized a dual-signal output strategy based on ferrocene (Fc) and PdPt@PCN-224 to enhance sensitivity and avoid false positive results. PdPt@PCN-224 nanocomposites exhibited strong catalytic activity towards H_2_O_2_ reduction, significantly improving detection sensitivity. The presence of CEA caused a decrease in the H_2_O_2_ reduction current and an increase in the Fc oxidation current, enabling accurate CEA quantification. Under optimal conditions, the aptasensor demonstrated a wide linear range from 1 pg/mL to 100 ng/mL, with low LOD of 0.98 pg/mL using Fc and PdPt@PCN-224 as signal labels. The platform showed excellent reproducibility with RSDs of 4.28% (Fc) and 3.30% (H_2_O_2_) for six biosensors. Table 4 shows the key characteristics of SPE-based electrochemical sensors for the detection of CEA.

### 4.4. BRCA1

**Figure 11:**
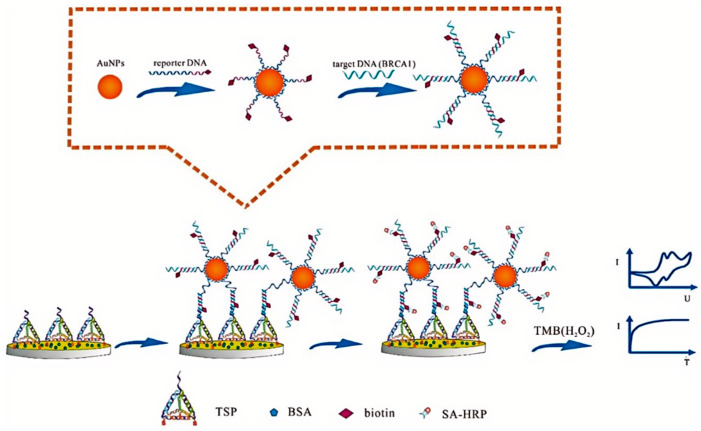
Scheme of fabrication of electrochemical DNA sensor for BRCA1 detection. CV curve and amperometry (IT curve) were applied to investigate the electrochemical behavior of the proposed electrochemical DNA sensor. Reprinted with permission from Ref. [81]. Copyright 2020 MDPI.

Genetic biomarkers, including specific gene mutations and alterations in gene expression patterns, play a crucial role in breast cancer risk assessment, diagnosis, and treatment planning. SPE-based biosensors have shown promising results in the detection of genetic markers, offering potential alternatives to traditional molecular biology techniques. The detection of BRCA1 gene mutations, which are associated with increased breast cancer risk, has been a focus of SPE-based genetic biomarker sensing. These approaches often involve the immobilization of specific DNA probes on the electrode surface, followed by hybridization with target DNA sequences from patient samples. Feng et al. [81] developed a sensitive electrochemical DNA sensor for detecting the breast cancer susceptibility gene BRCA1. The sensor utilized a DNA tetrahedral-structured probe (TSP) and Au-SPE. to achieve signal amplification. A sandwich system was formed by hybridizing capture DNA, target DNA (BRCA1), and reporter DNA (Figure 11). The sensor demonstrated a linear detection range from 1 fM to 1 nM, with a low detection limit of 0.1 fM. The sensor’s performance was attributed to the stable sandwich structure and signal enhancement from AuNPs-DNA reporter complexes. In another work, Li et al. [82] investigated the use of SWCNT-SPEs for electrochemical detection of DNA hybridization related to the BRCA1 breast cancer gene. The researchers optimized probe and target concentrations, finding 80 μg/mL and 120 μg/mL, respectively, as optimal. They achieved a detection limit of 378.52 nM for the target DNA. Electrochemical impedance spectroscopy complemented the voltammetric results, showing a 25.4% decrease in charge transfer resistance after DNA hybridization. The combination of SWCNTs and SPE proved effective for monitoring DNA hybridization, with potential applications in detecting BRCA1 mutations in biological samples.

### 4.5. MicroRNAs

**Figure 12:**
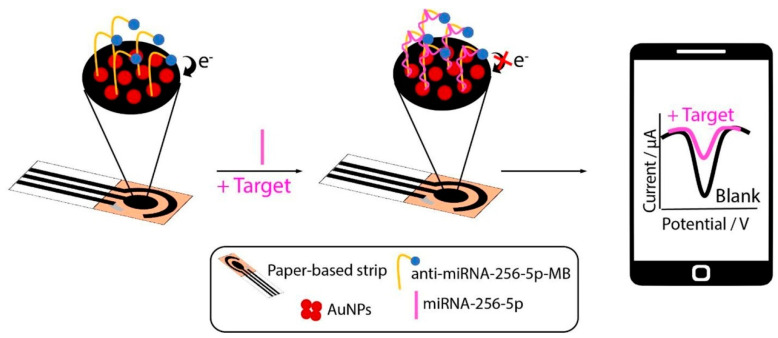
Scheme of paper-based SPE to detect miRNA-652. More details can be found in the text. Reprinted with permission from Ref. [83]. Copyright 2024 Elsevier.

MicroRNAs (miRNAs) have emerged as important genetic biomarkers in breast cancer, with specific miRNA expression profiles associated with different cancer subtypes and disease stages. SPE-based biosensors for miRNA detection often exploit the high specificity of nucleic acid hybridization combined with signal amplification strategies to achieve the necessary sensitivity for these low-abundance targets. For example, Raucci et al. [83] developed a paper-based electrochemical biosensor for detecting miRNA-652, a biomarker associated with triple-negative breast cancer (TNBC). The device consisted of an SPE on office paper, modified with AuNPs and an anti-miRNA probe (Figure 12). After optimizing experimental parameters, the biosensor demonstrated a detection limit of approximately 0.4 nM for miRNA-652 in both standard solutions and human serum. The system exhibited satisfactory repeatability of about 5% and good selectivity against other potential interfering miRNAs. To enhance sensitivity, the researchers introduced an innovative pre-concentration step using external wax-patterned chromatographic paper disks. This approach improved the detection limit by 10-fold with just 10 pre-concentration steps, applicable to both standard and human serum samples. The integration of the office paper-based SPE with the external chromatographic paper-based disk for pre-concentration resulted in a disposable device capable of providing immediate feedback in liquid biopsy applications. Another electrochemical biosensor was developed for the simultaneous detection of multiple miRNAs related to breast cancer [84]. The sensor utilized an SPE modified with reduced graphene oxide, poly(2-aminobenzylamine), and AuNPs to enhance sensitivity. Porous hollow Ag-Au nanoparticles tagged with metal ions were used as labels. An anti-DNA-RNA hybrid antibody enabled detection of different hybridized capture DNAs and miRNAs. The biosensor exhibited high selectivity, stability, and sensitivity, with a wide linear range from 1 fM to 10 nM. Detection limits were 0.98 fM, 3.58 fM, and 0.25 fM for miRNA-155, miRNA-21, and miRNA-16, respectively. The platform demonstrated good specificity, with one-base mismatched miRNA-21 detected at only 39.5% of the signal, three-base mismatched at 3.2%, and completely mismatched similar to blank levels.

### 4.6. Metabolite Biomarkers

Metabolite biomarkers offer a unique perspective on the biochemical changes associated with breast cancer, reflecting alterations in cellular metabolism that occur during tumor development and progression. SPE-based biosensors for metabolite detection often incorporate enzymes or other biocatalysts to achieve high specificity and sensitivity. Sarcosine, a metabolite that has been linked to breast cancer aggressiveness, has been successfully detected using an SPE-based biosensor incorporating sarcosine oxidase [85]. This enzyme catalyzes the oxidation of sarcosine, producing hydrogen peroxide as a byproduct. The amperometric detection of hydrogen peroxide provides a quantitative measure of sarcosine concentration. Another metabolite of interest in breast cancer is glutamate, which plays a role in tumor cell proliferation and invasion. An SPE-based biosensor for glutamate detection utilizes glutamate oxidase immobilized on porous Co_3_O_4_ nanocubes [86]. The enzyme catalyzes the oxidation of glutamate, generating hydrogen peroxide, which is then electrochemically detected. This system demonstrates high specificity for glutamate in the presence of other amino acids and has shown promise for monitoring glutamate levels in breast cancer cell cultures.

## 5. Challenges and Future Perspectives

Despite the significant advancements in SPE-based breast cancer biosensors, several challenges remain that limit their widespread adoption in clinical settings. One of the primary limitations is the complexity of biological samples, which can interfere with sensor performance. The presence of numerous proteins, metabolites, and other biomolecules in blood or tissue samples can lead to non-specific binding and false positive results [87]. Additionally, the low concentration of certain biomarkers, particularly in early-stage cancer, poses a significant challenge for detection sensitivity. Current SPE-based biosensors often struggle to achieve the ultra-low detection limits required for early cancer diagnosis without complex sample preparation or signal amplification strategies [88]. Another limitation is the stability and reproducibility of SPE-based biosensors over time. The surface modifications and biorecognition elements used in these sensors can degrade or lose activity during storage or repeated use, affecting the reliability of results. Furthermore, batch-to-batch variations in electrode production and modification processes can lead to inconsistencies in sensor performance, hindering their potential for standardized clinical use.

To address these challenges, research is focused on developing novel materials and fabrication approaches. Emerging nanomaterials, such as two-dimensional transition metal dichalcogenides and metal-organic frameworks, offer unique properties that could enhance sensor sensitivity and stability [89]. These materials provide high surface areas for biomarker capture and possess intrinsic catalytic activities that can amplify detection signals. While nanomaterials offer many advantages in enhancing sensor sensitivity and specificity, it is important to acknowledge that they can also contribute to variability issues. The inherent batch-to-batch variations in nanomaterial synthesis and properties may exacerbate inconsistencies in electrode structure and performance. This variability poses a significant challenge for industrial-scale production and standardization of nanomaterial-enhanced SPEs. Several approaches should be explored. One strategy is the development of robust calibration protocols specifically tailored for nanomaterial-based electrodes. These protocols may involve the use of well-characterized reference materials and multi-point calibration curves to account for variations in nanomaterial properties. Additionally, the integration of in-situ calibration features into sensor designs is being investigated. This approach allows for real-time adjustment and compensation for batch-to-batch differences, potentially improving the reliability of measurements in clinical settings. It is also important to address the apparent discrepancy between the use of precious metals like gold in SPEs and claims of low-cost production. While gold pastes for screen printing can indeed be expensive, alternative approaches are being explored to balance performance and cost-effectiveness. For example, sputtered gold thin-film electrodes have gained traction in industrial practice for POC systems, offering a more economical solution for large-scale production while maintaining excellent conductivity and biocompatibility.

Advanced fabrication techniques, including 3D printing and laser-induced graphene formation, are being explored to create more precise and reproducible electrode structures with enhanced electrochemical properties [90]. The integration of SPE-based biosensors with microfluidic systems represents a promising avenue for overcoming sample complexity issues. Microfluidic devices can incorporate sample preparation steps, such as filtration and preconcentration, directly on-chip, reducing interference from complex matrices and improving detection sensitivity. Moreover, the combination of microfluidics with SPEs enables the development of fully integrated lab-on-a-chip devices capable of performing multiple analytical steps in a single, compact platform.

While SPE-based biosensors for breast cancer biomarkers have shown great promise in laboratory settings, their translation to industrial applications and clinical trials is still in progress. Currently, most of the research in this field is at the proof-of-concept or early validation stage, where the focus is on demonstrating the feasibility and potential of these sensors in controlled environments. The transition from laboratory research to clinical application is a complex and time-consuming process, requiring extensive validation, regulatory approval, and collaboration between researchers, clinicians, and industry partners. At present, the majority of studies involving SPE sensors for breast cancer biomarkers are conducted as collaborative efforts between analytical chemists and medical professionals, using patient blood samples to assess the potential real-world applicability of these devices.

## 6. Conclusions

In conclusion, this review has highlighted the significant potential of SPEs as low-cost, sensitive, and versatile platforms for breast cancer biomarker detection. SPEs offer numerous advantages, including simple fabrication, disposability, and compatibility with various modification strategies to enhance performance. The integration of nanomaterials like carbon nanotubes, graphene, and metal nanoparticles has dramatically improved the sensitivity and specificity of SPE-based biosensors for detecting crucial breast cancer biomarkers such as HER2, CA15-3, and CEA. Novel approaches in biomolecule immobilization, including the use of aptamers and molecularly imprinted polymers, have further expanded the capabilities of these sensors. The development of multiplexed assays on SPEs has enabled simultaneous detection of multiple biomarkers, providing a more comprehensive assessment of breast cancer status. While significant progress has been made, challenges remain in terms of sensor stability, reproducibility, and performance in complex biological matrices. Emerging research directions, such as the integration of SPEs with microfluidic systems and the development of wearable sensors, offer promising avenues for overcoming these limitations. The potential for early screening and therapy monitoring using SPE-based biosensors is substantial, with the possibility of detecting cancer biomarkers at much earlier stages and enabling more timely treatment adjustments. However, the path from laboratory development to clinical implementation involves rigorous validation studies and regulatory considerations. As sensor technologies continue to advance, SPE-based biosensors are poised to play a crucial role in improving breast cancer diagnostics and patient care, particularly in resource-limited settings and for POC applications.

## Figures and Tables

**Table 1:** Common breast cancer biomarkers, their characteristics, and clinical utility.

Biomarker Name	Size	Utility	Level in Serum (Normal/Pathological)
BRCA1	5592 nt	Diagnostic for BC	N/A (genetic test)
BRCA2	11,385 nt	Diagnostic for BC	N/A (genetic test)
HER2	2200 nt	Diagnostic and prognostic for BC	<15 ng/mL/≥15 ng/mL [5]
CA15-3	300–450 kDa	Prognostic for BC	<30 U/mL/>30 U/mL [6]
CEA	180–200 kDa	Important for BC treatment strategy	<5 ng/mL/>5 ng/mL [7]
CA125	200–2000 kDa	Prognostic for BC	<35 U/mL/>35 U/mL [8]
MUC1	2000 nt	Diagnostic and prognostic for BC	<30 U/mL/≥30 U/mL [9]
VEGF	14,000 nt	Important for BC treatment strategy	<291 pg/mL/≥321.4 pg/mL [10]
CTC	N/A	Prognostic and important in therapy monitoring of BC	0–1 CTC/7.5 mL/>2 CTC/7.5 mL [11]
miRNA-21	20–24 nt	Diagnostic for BC	N/A (expression levels compared to controls)
miRNA-16	20–24 nt	Diagnostic for BC	N/A (expression levels compared to controls)
miRNA-155	21–24 nt	Diagnostic for BC	N/A (expression levels compared to controls)
miRNA-652	22 nt	Diagnostic for BC	N/A (expression levels compared to controls)

**Table 2:** Key characteristics of SPE-based electrochemical sensors for the detection of HER2.

Modification Components	Linear Detection Range	Limit of Detection	Total Assay Time	Automation/Manual Steps	Real Sample	Reference
MWCNTs+ MUC1 aptamers	100 to 2000 ng/mL	20 ng/mL	Not explicitly stated	Incubate the aptamer-modified electrode with 50 μL of the sample containing MUC1 for 45 min. Rinse the electrode thoroughly with distilled water to remove unbound analytes. Perform EIS measurement using a potentiostat/galvanostat instrument.	Human blood serum	[45]
SPCE/CBAmN3/anti-HER2	-	0.005 ng/mL	Not explicitly stated	Apply the serum sample containing HER2 to the prepared biosensor surface. Allow HER2 to bind to the immobilized anti-HER2 antibodies on the sensor surface. Perform EIS measurements. This involves: For glycoprofiling, apply lectins (such as PHA-E) to the captured HER2. Perform another EIS measurement after lectin binding.	Human serum	[52]
DNA aptamer/MCH	0.001 to 100 ng/mL	0.179 ng/mL	Not explicitly stated	Incubate the modified SPEs with the sample containing HER2 for 30 min. Wash the electrodes with PBS buffer. Perform EIS measurement in PBS containing 2.5 mM redox probe. Analyze the change in Rct to determine the HER2 concentration.	Human serum	[54]
bi-SPCE-AuNP-Ab	9.8 to 50 ng/mL	2.9 ng/mL	Not explicitly stated	Place 60 μL of a mixture containing the antigens (HER2-ECD and CA 15-3) and anti-CA15-3-bio antibody on the sensor and incubate for 1 h. Wash the sensor with buffer 1. Add 60 μL of anti-HER2-bio antibody solution and incubate for 30 min. Wash the sensor with buffer 1. Add 60 μL of streptavidin-alkaline phosphatase (S-AP) solution and incubate for 1 h. Wash the sensor with buffer 2. Add 60 μL of 3-indoxyl phosphate/silver nitrate solution and allow the enzymatic reaction to proceed for 20 min. Perform linear sweep voltammetry from −0.02 to 0.4 V at 50 mV/s to measure the electrochemical oxidation current of the enzymatically deposited silver.	-	[55]
MB-Ab1/HER2/Ab2-biotin-SA-AP/SPE	0 to 15 ng/mL	6 ng/mL	Not explicitly stated	Mix the functionalized magnetic beads with the sample containing HER2 and incubate for 60 min. Wash the beads three times using a magnetic separator. Add the secondary antibody solution and incubate for 20 min. Wash the beads again three times. Incubate with streptavidin-alkaline phosphatase conjugate for 10 min. Wash the beads twice. Resuspend the beads in buffer and place on the screen-printed electrode. Add the enzyme substrate (1-naphthyl phosphate). Perform DPV measurement after 5 min.	Serum sample	[56]
AuNPs/Aptamer/MCH	0.001 to 100 ng/mL	0.001 ng/mL	Not explicitly stated	Cast 8 μL of various concentrations of HER2 solution onto the prepared SPE/AuNPs/Aptamer/MCH electrode surface. Incubate at room temperature for 5 min. Wash with PBS to remove any unbound HER2. Allow a binding reaction time of 10 min at 25 °C. Perform DPV measurements to detect the HER2 concentration.	-	[24]
MPA/Cys/AuNP@HER2 aptamer	0.01 to 15.0 ng/mL; 15.0 to 100.0 ng/mL	1.52 ng/mL	Not explicitly stated	Incubate the fabricated aptasensor with different concentrations of HER2 protein solution for 60 min at room temperature. Wash the sensor with PBS buffer. Perform electrochemical measurement using DPV in 10 mM [Fe(CN)_6_]^3−/4−^ redox probe solution. Record the voltammogram from −0.2 to +0.4 V with a scan rate of 50 mV/s. Analyze the reduction in current peak corresponding to increasing HER2 concentrations	-	[58]
PLLF-RNA aptamer	10 to 60 ng/mL.	3 ng/mL	4 min for detection	Deplete albumin from the serum sample using an albumin depletion kit. Add the prepared serum sample to the aptamer-modified working electrode surface. Allow the binding reaction to occur at 25 °C for 30 min. Add MB to the modified electrode and incubate for 5 min at 25 °C. Wash with Tris-HCl buffer to remove unbound molecules. Perform DPV measurement in Tris-HCl buffer for 4 min.	Human serum	[59]
ELISA-HER2	5–20 ng/mL; 20–200 ng/mL	4 ng/mL	Not explicitly stated	Pipette 20 μL of the prepared HER2 antigen sample onto the working electrode of the SPE Incubate for 2 h at room temperature Wash the electrode three times with 50 μL of wash buffer solution and dry for 10 min at room temperature Add 20 μL of detection antibody and incubate for 2 h at room temperature Wash and dry the electrode again as in step 3 Add 20 μL of streptavidin-HRP and incubate for 0.3 h (18 min) at room temperature Wash and dry the electrode again Add 20 μL of TMB-H_2_O_2_ (1:1 mixture) and incubate for 0.3 h (18 min). Add 50 μL of stop solution to stop the oxidation process. After 5 min, perform CV to record the electrochemical response.	Human serum	[57]
MPA/anti-HER-2/biotin-anti-HER-2/poly-HRP	-	0.012 ng/mL	15 min	Insert the prepared electrochemical array chip into the microfluidic device. Flush the array with 0.05% PBS-T-20 for 2 min at a flow rate of 200 μL/min. In a 1 mL centrifuge tube, mix: - 100 μL of optimized secondary antibody-HER-2 - 100 μL of optimized poly-HRP dilution (1:250 in 0.1% BSA) - 100 μL of sample Immediately inject this mixture into the microfluidic channel housing the electrochemical array chips at a flow rate of 200 μL/min. Allow to incubate for 10 min. Wash with 0.05% PBS-T20 for 2 min. Pass a solution of 1 mM hydroquinone through the microfluidic channel. Inject a mixture of 100 mM H_2_O_2_ and 1 mM HQ in PBS. Generate an amperometric signal at −0.15 V vs. pseudo-Ag/AgCl.	Pooled human serum	[60]
AuNP-anti-HER2	15 to 100 ng/mL	4.4 ng/mL	2 h and 50 min	Incubate the sample containing HER2 ECD with the biotinylated detection antibody for 5 min. Place the mixture on the prepared sensor surface (which has the capture antibody already immobilized) and incubate for 1 h. Add S-AP solution and incubate for 1 h. Wash the sensor with Buffer 2 and dry. Add a solution containing the enzymatic substrate (3-indoxyl phosphate, 3-IP) and silver ions, and incubate for 20 min. Perform linear SWV to detect the analytical signal (potential scan from −0.03 to +0.4 V at a scan rate of 0.05 V/s).	Human serum	[61]
MWCNT/AuNP	7.5–50 ng/mL	0.16 ng/mL	2 h and 20 min	Incubate a mixture containing the detection antibody, HER2-ECD sample, and BSA on the modified electrode surface for 30 min. Wash the electrode with buffer 1. Add S-AP solution and incubate for 60 min. Wash the electrode first with buffer 1, then with buffer 3. Add a mixture containing the enzymatic substrate (3-indoxyl phosphate) and silver nitrate to the electrode surface and incubate for 20 min. Perform LSV to obtain the electrochemical signal.	Human serum	[62]
Affibody/AuNPs	0 to 40,000 ng/L	6000 ng/L	Not explicitly stated	Incubate the prepared affisensor with the sample containing HER2 protein for 60 min at room temperature. Rinse the affisensor with 0.1 M PBS buffer pH 7.4 solution. Perform EIS measurements using 0.01 M [Fe(CN)_6_]^3−/4−^ as redox probe prepared in PBS buffer (0.1 M, pH 7.4). Analyze the changes in Rct values obtained after the affinity reaction with HER2 to determine the concentration.	Human serum	[63]
AuNP-Ab-HRP	0.25 to 50 ng/mL	0.03 ng/mL	Not explicitly stated	Apply a sample containing the target biomarker (HER-1 or HER-2) to the sensor surface. Allow time for the antigen in the sample to bind to the capture antibodies on the sensor. Add the detector antibody and allow it to bind. Add the enzyme-labeled secondary antibody (either anti-species HRP or AuNP-Ab-HRP conjugate). Add the substrate solution (TMB/H_2_O_2_). Perform chronoamperometric measurement at −200 mV for 110 s. Analyze the resulting current signal to determine the biomarker concentration.	Human serum	[64]
Strept-MB/Biot-Af	0 to 20 ng/mL	1.8 ng/mL	Not explicitly stated	Incubation of the functionalized magnetic beads with the HER2 protein sample for 20 min. Washing steps to remove unbound proteins. Incubation with the secondary biotinylated affibody for 45 min. Washing steps. Incubation with streptavidin-alkaline phosphatase enzyme for 10 min. Washing steps. Resuspension of the beads in buffer. Placing the bead suspension onto the working electrode of the screen-printed electrochemical cells. Addition of the enzyme substrate (1-naphthyl phosphate). Incubation for 6 min. Performing differential pulse voltammetry measurements.	Human serum	[65]
ABA/anti-HER2	0.005 to 0.04 ng/mL	0.005 ng/mL	Not explicitly stated	Place the biofunctionalized SPE chip into the microfluidic system. Introduce the sample (either PBS containing HER2 or real saliva) into the microfluidic system. Perform EIS measurements using the following parameters: Analyze the EIS data using the EIS spectrum analyzer software to fit the results to the equivalent circuit model. Calculate the normalized resistance change (ΔR3/R3) based on the fitted data.	Human saliva	[66]

**Table 3:** Key characteristics of SPE-based electrochemical sensors for the detection of CA 15-3.

Modification Components	Linear Detection Range	Limit of Detection	Total Assay Time	Automation/Manual Steps	Real Sample	Reference
Ab2-HRP/CA15-3/BSA/Ab1/MPA/Au-rGO	1 × 10^−9^ to 1 × 10^4^ U/mL	8 × 10^−10^ U/mL	Not explicitly stated	Incubate the prepared immunosensor with 15 µL of CA15-3 solution for 30 min at 20 °C. Add 10 µL of anti-CA15-3 conjugated with HRP (Ab2, 1 µg/mL) and incubate for 30 min to form sandwich immune-complexes. Perform SWV measurement in PBS solution (pH 7.4) containing 2.5 mM HQ and 2.5 mM H_2_O_2_. Record the SWV from 0.3 to −0.25 V, with step potential −5 mV, amplitude 75 mV, and frequency 2 Hz. Analyze the resulting voltammogram to determine the CA15-3 concentration based on the calibration curve.	Artificial saliva	[25]
HRP-MBs-Ab2/CA 15-3/Ab1-P(1,5DAN)/PPy NWs	0.05 to 20 U/mL	0.02 U/mL	Not explicitly stated	Incubate the HRP-MBs-Ab2/CA 15-3 complexes with the immunosensor (Ab1–P(1,5DAN)/PPy NWs/SPEs) for 60 min. Place 50 μL of PBS containing a mixture solution of 0.1 mM H_2_O_2_ and 1 mM HQ onto the electrode surface. Perform electrochemical measurements (CV or DPV) at room temperature. Analyze the resulting voltammograms to determine the CA 15-3 concentration based on the calibration curve.	-	[50]
MIP	5 to 50 U/mL	1.5 U/mL	15 min	Incubate the MIP/Au-SPE sensor in the CA 15-3 standard or sample solution for 10 min. After incubation, perform a DPV measurement using a 5 mM [Fe(CN)_6_]^3−/4−^ solution as the redox probe. Measure the peak current intensity from the DPV voltammogram. Compare the peak current intensity to a calibration curve to determine the CA 15-3 concentration. Regenerate the sensor by extracting CA 15-3 from the polymer using 0.5 M oxalic acid solution.	Human serum	[51]
bi-SPCE-AuNP-Ab	17 to 70 U/mL	5.0 U/mL	Not explicitly stated	Place 60 μL of a mixture containing the analytes (HER2-ECD and CA 15-3) and anti-CA15-3-bio antibody on the sensor and incubate for 1 h. Wash the sensor with buffer 1. Add 60 μL of anti-HER2-bio antibody solution and incubate for 30 min. Wash the sensor with buffer 1. Add 60 μL of S-AP solution and incubate for 1 h. Wash the sensor with buffer 2. Add 60 μL of 3-indoxyl phosphate/silver nitrate solution and incubate for 20 min. Perform linear sweep voltammetry between −0.02 and 0.4 V at 50 mV/s to measure the electrochemical oxidation current of the enzymatically deposited silver.	-	[55]
AuNP/Ab	2 to 16 U/mL	0.56 U/mL	1 h	Add 30 µL of the sample solution containing CA 15-3 to the sensor. Incubate the sensor with the sample for 1 h at 4 °C. Wash the sensor with 0.10 M phosphate-buffered saline (PBS, pH 7.5). Dry the sensor under nitrogen. Perform electrochemical measurements using DPV with 0.50 mM potassium ferrocyanide in PBS as the redox species.	Human saliva and serum	[67]
BSA/Ab/MWCNT-dP2ClAn	5 to 100 U/mL	0.66 U/mL	Not explicitly stated	The prepared BSA/Ab/MWCNT-dP2ClAn/SPE electrode is incubated with the sample containing CA15-3 for 40 min at 25 °C. This incubation time was determined to be optimal for binding between the antibody and the CA15-3 antigen. Washing: After incubation, the electrode is washed three times with 6 µL of PBS (0.01 M, pH 7.4) to remove any unbound molecules. Measurement: The detection is performed using DPV in a solution of 5 mM K_3_Fe(CN)_6_/K_4_Fe(CN)_6_ containing 0.1 M KCl. Analysis: The current response is measured and compared to the calibration curve to determine the CA15-3 concentration.	Human serum	[68]
CNE/AuNP/MIP	5 to 35 U/mL	1.16 U/mL	Not explicitly stated	The sample containing CA 15-3 is manually dropped onto the sensor and incubated for 1 h at 4 °C. After incubation, the electrochemical measurement is performed using chronoamperometry. The current response is then analyzed to determine the CA 15-3 concentration based on the calibration curve.	Human serum	[69]
C-PDDA-AuNPs-Ab1-BSA	0.01 to 1 U/mL	0.006 U/mL	Not explicitly stated	Off-line biomarker capture: - Add 20 μL of MP-Ab2-HRP to 340 μL of buffer containing CA15-3 - Incubate for 30 min at 37 °C - Magnetically separate and wash the captured analyte three times - Resuspend in 125 μL of buffer On-line detection in the DμID: - Inject the resuspended sample into the microfluidic system - Allow incubation time (70 min based on optimization) - Wash the microfluidic channel with PBS-BSA - Inject a mixture of 0.1 mM H_2_O_2_ and 1.0 mM HQ - Perform amperometric detection	Human serum	[70]
PoPDA/Au	0.25 to 10.00 U/m	0.05 U/mL	Less than 30 min	Incubate the sensor with the sample containing CA15-3 for 15 min. Perform electrochemical measurements, specifically SWV or EIS. Analyze the electrochemical response to determine the CA15-3 concentration.	Human serum	[71]
MIP	0.10 to 100 U/mL	0.10 U/mL	Not explicitly stated	Incubate the MIP sensor surface with the sample containing CA 15-3 for 30 min. Wash the sensor surface with water to remove any loosely bound materials. Replace the sample solution with a drop of 5 mM [Fe(CN)_6_]^3−/4−^ redox probe solution. Perform DPV measurements to detect the CA 15-3 concentration.	Human serum	[72]
AMPTMA-MIP	0.001 to 100 U/mL	0.000909 U/mL	Less than 20 min	Prepare the sample solution containing CA 15-3 in PB at pH 5.8. Apply 5 μL of the prepared sample solution to the working electrode surface of the MIP sensor. Incubate the sample on the sensor surface for 20 min. After incubation, perform electrochemical measurements using SWV with a redox probe solution containing 5.0 mM K_3_[Fe(CN)_6_] and 5.0 mM K_4_[Fe(CN)_6_] in phosphate buffer. Record the current response using the following SWV parameters: step potential of 5 mV, pulse amplitude of 10 mV, and frequency of 5 Hz. Analyze the current response to determine the CA 15-3 concentration based on the calibration curve.	Human serum	[73]
CoS_2_-GR-AuNPs	0.1 to 150 U/mL	0.03 U/mL	Not explicitly stated	Incubate the prepared immunosensor with different concentrations of CA15-3 antigen for 3 h at 4 °C. Rinse the immunosensor with deionized water to remove any unstable adsorbed CA15-3 from the electrode surface. Perform electrochemical measurements using 1.0 mM catechol as a probe. Measure the DPV response, which decreases with increasing CA15-3 concentration.	Human serum	[74]

**Table 4:** Key characteristics of SPE-based electrochemical sensors for the detection of CEA.

Modification Components	Linear Detection Range	Limit of Detection	Total Assay Time	Automation/Manual Steps	Real Sample	Reference
POct-Ab-CEA	1.5 × 10^−5^ to 1.5 ng/mL	1.36 × 10^−4^ ng/mL	Not explicitly stated	Add 10 μL of sample containing CEA to the sensor surface. Incubate for 20 min at room temperature in a moist chamber. Rinse the electrodes with 100 mM PBS pH 7.1 to remove unbound analyte. Perform EIS measurement. This is performed in 100 mM PBS pH 7.1 plus equal ratio of 10 mM K_3_[Fe(CN)_6_]/K_4_[Fe(CN)_6_]. Record impedance data from 2.5 KHz to 0.25 Hz with a modulation voltage of 10 mV at 0 V applied potential relative to the reference.	human serum	[77]
POct-Aff-CEA	1.5 × 10^−5^ to 1.5 × 10^−3^ ng/mL	1.76 × 10^−4^ ng/mL	Not explicitly stated	Same as above	Human serum	[77]
Ab1/AuNRs-PWE/Au/BSA-metal ion-Ab2	1 × 10^−4^ to 50 ng/mL	8 × 10^−5^ ng/mL	Not explicitly stated	Drop 2.0 µL of sample solution containing different concentrations of CEA and CA125 onto the AuNRs-PWE. Incubate for 210 s at room temperature. Wash with PBS (pH 7.4). Add 4.0 µL of the designed bioconjugates (Ab2–Au/BSA–metal ion) to the AuNRs-PWE. Incubate for 210 s at room temperature. Wash with PBS (pH 7.4). Fold the device using a simple homemade device-holder. Fix and connect the 3D origami EC immunodevice to the electrochemical workstation. Add 40 µL of HAc/NaAc solution (pH 4.5) as the supporting electrolyte. Perform a DPV scan from −0.9 V to −0.3 V to record the amperometric responses for simultaneous detection of CEA and CA125.	Human serum	[75]
HRP-Ab2/CEA/Ab1/AuNPs/rGO	0.5 to 25 ng/mL; 250 to 2000 ng/mL	0.28 ng/mL	Not explicitly stated	Incubation of the sensor with the sample containing CEA for a specific time (duration not specified). Washing steps to remove unbound analytes. Addition of secondary antibody labeled with HRP and incubation (time not specified). Another washing step to remove unbound secondary antibodies. Addition of H_2_O_2_ substrate (5 μM in 0.1 M PBS) for the electrochemical measurement. Performing CV or amperometry to measure the electrochemical response.	-	[46]
HRP-Ab2/CEA/Ab1/AgNPs-rGO	50 to 500 ng/mL	35 ng/mL	Not explicitly stated	Add diluted CEA solution (antigen) to the modified SPE and incubate for 2–3 h at room temperature. Wash the electrode twice with PBS to remove unbound antigen. Add secondary detection antibody and incubate for 2 h at room temperature. Wash the electrode four times with PBS to remove unbound antibody. Add HRP-conjugated secondary antibody and incubate for 30 min to 2 h at room temperature. Wash the electrode four times with PBS to remove unbound conjugated antibody. Add enzyme substrate (H_2_O_2_) using a pipette. Perform electrochemical measurement (likely using cyclic voltammetry, though the specific measurement step is not explicitly detailed for the final detection).	-	[47]
GCE/Au NPs/Ab1/BSA/CEA/Ab2-Ag NPs@CS-Hemin/rGO	2 × 10^−5^ to 200 ng/mL	6.7 × 10^−6^ ng/mL	Not explicitly stated	Add the sample containing CEA to the prepared immunosensor electrode and incubate for 1 h at 4 °C. Rinse the electrode with PBS (pH 7.0) to remove unbound CEA. Add the Ab2 label (Ag NPs@CS-Hemin/rGO-Ab2) to the electrode surface and incubate for 45 min at 4 °C. Rinse the electrode again with PBS (pH 7.0) to remove unbound Ab2 label. Place the electrode in 10.0 mL of PBS (pH 7.0). Perform amperometric i-t curve measurement at a potential of −0.4 V. Once the background current stabilizes, inject 10.0 μL of H_2_O_2_ (5.0 mol/L) into the PBS under mild stirring. Record the change in current signal.	Human serum	[49]
PdPt@PCN-224-P-dsDNA/Au	0.001 to 100 ng/mL	9.8 × 10^−4^ ng/mL	Not explicitly stated	Drop different concentrations of CEA onto the PdPt@PCN-224-P-dsDNA/Au/SPE. Incubate the electrode with CEA at 37 °C for 30 min. Rinse the electrode with purified water. Immerse the electrode in PBS buffer containing 10 mM H_2_O_2._ Perform electrochemical measurements using the smartphone-based platform. Use the PSTouch software on the smartphone to conduct and analyze the electrochemical measurements.	Human serum	[76]
CEA-MWCNT-PEI	0.005 to 500 ng/mL	0.001 ng/mL	Not explicitly stated	Incubate the αCEA immobilized MWCNT-PEI/SPE with 50 μL of the CEA standard or sample for 30 min at 25 ± 3 °C. Wash the electrode with PBS (0.1 M, pH 7.0). Incubate the electrode with 10 μL of αCEA-FCL in 100 μL PBS (0.01 M, pH 7.0) for 30 min at 25 ± 3 °C. Wash the electrode again with PBS (0.1 M, pH 7.0). Add 100 μL of methanolic solution of Triton X (2%) in PBS (0.1 M, pH 7.0) to dissolve the bound FCL. Perform SWV measurement from −0.3 to 0.5 V with 4 mV potential steps, 25 Hz frequency, and 50 mV amplitude.	Human serum	[78]
P-SPGE/R1/anti-CEA	1.0 to 100.0 ng/mL	0.33 ng/mL	Not explicitly stated	Incubate the prepared electrode in the CEA solution for 30 min. Wash the electrode with PBS (phosphate-buffered saline). Perform DPV measurements in PBS (10 mM, pH 7.4). Record the DPV signals by scanning from −0.90 to +0.20 V at a pulse amplitude of 50 mV and a scan rate of 10 mV/s.	Human serum	[79]
NH2-G/Thi/AuNPs-SPWE/Anti-CEA	0.05 to 500 ng/mL	0.01 ng/mL	Not explicitly stated	Apply the sample containing CEA to the sample tab of the paper-based device. Allow the sample to flow through the microfluidic channel to the detection zone containing the modified working electrode. Perform DPV measurement using the three-electrode system (working, counter, and reference electrodes) printed on the paper device. Measure the peak current from the DPV response. Calculate the CEA concentration using the calibration curve relating peak current to log CEA concentration.	Human serum	[80]

## Data Availability

Data are contained within the article.

## References

[1] Coleman C. (2017). Early Detection and Screening for Breast Cancer. Semin. Oncol. Nurs..

[2] Ginsburg O., Yip C.-H., Brooks A., Cabanes A., Caleffi M., Dunstan Yataco J.A., Gyawali B., McCormack V., McLaughlin de Anderson M., Mehrotra R. (2020). Breast Cancer Early Detection: A Phased Approach to Implementation. Cancer.

[3] Levenson V.V. (2007). Biomarkers for Early Detection of Breast Cancer: What, When, and Where?. Biochim. Biophys. Acta (BBA)-Gen. Subj..

[4] Li J., Guan X., Fan Z., Ching L.-M., Li Y., Wang X., Cao W.-M., Liu D.-X. (2020). Non-Invasive Biomarkers for Early Detection of Breast Cancer. Cancers.

[5] Fehm T., Becker S., Duerr-Stoerzer S., Sotlar K., Mueller V., Wallwiener D., Lane N., Solomayer E., Uhr J. (2007). Determination of HER2 Status Using Both Serum HER2 Levels and Circulating Tumor Cells in Patients with Recurrent Breast Cancer Whose Primary Tumor Was HER2 Negative or of Unknown HER2 Status. Breast Cancer Res..

[6] Ryu J.M., Kang D., Cho J., Lee J.E., Kim S.W., Nam S.J., Lee S.K., Kim Y.J., Im Y.-H., Ahn J.S. (2023). Prognostic Impact of Elevation of Cancer Antigen 15-3 (CA15-3) in Patients with Early Breast Cancer with Normal Serum CA15-3 Level. J. Breast Cancer.

[7] Shao Y., Sun X., He Y., Liu C., Liu H. (2015). Elevated Levels of Serum Tumor Markers CEA and CA15-3 Are Prognostic Parameters for Different Molecular Subtypes of Breast Cancer. PLoS ONE.

[8] Fang C., Cao Y., Liu X., Zeng X.-T., Li Y. (2017). Serum CA125 Is a Predictive Marker for Breast Cancer Outcomes and Correlates with Molecular Subtypes. Oncotarget.

[9] Moreno M., Bontkes H.J., Scheper R.J., Kenemans P., Verheijen R.H.M., von Mensdorff-Pouilly S. (2007). High Level of MUC1 in Serum of Ovarian and Breast Cancer Patients Inhibits huHMFG-1 Dependent Cell-Mediated Cytotoxicity (ADCC). Cancer Lett..

[10] Reeves K.W., Ness R.B., Stone R.A., Weissfeld J.L., Vogel V.G., Powers R.W., Modugno F., Cauley J.A. (2009). Vascular Endothelial Growth Factor and Breast Cancer Risk. Cancer Causes Control.

[11] Thery L., Meddis A., Cabel L., Proudhon C., Latouche A., Pierga J.-Y., Bidard F.-C. (2019). Circulating Tumor Cells in Early Breast Cancer. JNCI Cancer Spectr..

[12] Ambrosi A., Airò F., Merkoçi A. (2010). Enhanced Gold Nanoparticle Based ELISA for a Breast Cancer Biomarker. Anal. Chem..

[13] Zaha D.C. (2014). Significance of Immunohistochemistry in Breast Cancer. World J. Clin. Oncol..

[14] Oloomi M., Moazzezy N., Bouzari S. (2020). Comparing Blood versus Tissue-Based Biomarkers Expression in Breast Cancer Patients. Heliyon.

[15] Taleat Z., Khoshroo A., Mazloum-Ardakani M. (2014). Screen-Printed Electrodes for Biosensing: A Review (2008–2013). Microchim. Acta.

[16] García-Miranda Ferrari A., Rowley-Neale S.J., Banks C.E. (2021). Screen-Printed Electrodes: Transitioning the Laboratory in-to-the Field. Talanta Open.

[17] Moreira F.T.C., Ferreira M.J.M.S., Puga J.R.T., Sales M.G.F. (2016). Screen-Printed Electrode Produced by Printed-Circuit Board Technology. Application to Cancer Biomarker Detection by Means of Plastic Antibody as Sensing Material. Sens. Actuators B Chem..

[18] Ahmed M.U., Hossain M.M., Safavieh M., Wong Y.L., Rahman I.A., Zourob M., Tamiya E. (2016). Toward the Development of Smart and Low Cost Point-of-Care Biosensors Based on Screen Printed Electrodes. Crit. Rev. Biotechnol..

[19] Arduini F., Micheli L., Moscone D., Palleschi G., Piermarini S., Ricci F., Volpe G. (2016). Electrochemical Biosensors Based on Nanomodified Screen-Printed Electrodes: Recent Applications in Clinical Analysis. TrAC Trends Anal. Chem..

[20] Yamanaka K., Vestergaard M.C., Tamiya E. (2016). Printable Electrochemical Biosensors: A Focus on Screen-Printed Electrodes and Their Application. Sensors.

[21] Li M., Li Y.-T., Li D.-W., Long Y.-T. (2012). Recent Developments and Applications of Screen-Printed Electrodes in Environmental Assays—A Review. Anal. Chim. Acta.

[22] Suresh R.R., Lakshmanakumar M., Arockia Jayalatha J.B.B., Rajan K.S., Sethuraman S., Krishnan U.M., Rayappan J.B.B. (2021). Fabrication of Screen-Printed Electrodes: Opportunities and Challenges. J. Mater. Sci..

[23] Dhanapala L., Krause C.E., Jones A.L., Rusling J.F. (2020). Printed Electrodes in Microfluidic Arrays for Cancer Biomarker Protein Detection. Biosensors.

[24] Harahsheh T., Makableh Y.F., Rawashdeh I., Al-Fandi M. (2021). Enhanced Aptasensor Performance for Targeted HER2 Breast Cancer Detection by Using Screen-Printed Electrodes Modified with Au Nanoparticles. Biomed. Microdevices.

[25] Martins T.S., Bott-Neto J.L., Oliveira O.N., Machado S.A.S. (2021). A Sandwich-Type Electrochemical Immunosensor Based on Au-rGO Composite for CA15-3 Tumor Marker Detection. Microchim. Acta.

[26] Chakraborty B., Das A., Mandal N., Samanta N., Das N., Chaudhuri C.R. (2021). Label Free, Electric Field Mediated Ultrasensitive Electrochemical Point-of-Care Device for CEA Detection. Sci. Rep..

[27] Chiorcea-Paquim A.-M. (2023). Advances in Electrochemical Biosensor Technologies for the Detection of Nucleic Acid Breast Cancer Biomarkers. Sensors.

[28] Erdem A., Eksin E., Congur G. (2015). Indicator-Free Electrochemical Biosensor for microRNA Detection Based on Carbon Nanofibers Modified Screen Printed Electrodes. J. Electroanal. Chem..

[29] Randviir E.P., Brownson D.A., Metters J.P., Kadara R.O., Banks C.E. (2014). The Fabrication, Characterisation and Electrochemical Investigation of Screen-Printed Graphene Electrodes. Phys. Chem. Chem. Phys..

[30] Beitollahi H., Mohammadi S.Z., Safaei M., Tajik S. (2020). Applications of Electrochemical Sensors and Biosensors Based on Modified Screen-Printed Electrodes: A Review. Anal. Methods.

[31] Foster C.W., Kadara R.O., Banks C.E., Banks C.E., Foster C.W., Kadara R.O. (2016). Fabricating Screen-Printed Electrochemical Architectures: Successful Design and Fabrication. Screen-Printing Electrochemical Architectures.

[32] Foster C.W., Kadara R.O., Banks C.E., Banks C.E., Foster C.W., Kadara R.O. (2016). Quality Control/Quality Assurance Analysis of Electrochemical Screen-Printed Sensors. Screen-Printing Electrochemical Architectures.

[33] Rao V.K., Sharma M.K., Pandey P., Sekhar K. (2006). Comparison of Different Carbon Ink Based Screen-Printed Electrodes towards Amperometric Immunosensing. World J. Microbiol. Biotechnol..

[34] Chu Z., Peng J., Jin W. (2017). Advanced Nanomaterial Inks for Screen-Printed Chemical Sensors. Sens. Actuators B Chem..

[35] Pérez-Fernández B., Costa-García A., Muñiz A.D.L.E. (2020). Electrochemical (Bio)Sensors for Pesticides Detection Using Screen-Printed Electrodes. Biosensors.

[36] Ferreira L.M.C., Reis I.F., Martins P.R., Marcolino-Junior L.H., Bergamini M.F., Camargo J.R., Janegitz B.C., Vicentini F.C. (2023). Using Low-Cost Disposable Immunosensor Based on Flexible PET Screen-Printed Electrode Modified with Carbon Black and Gold Nanoparticles for Sensitive Detection of SARS-CoV-2. Talanta Open.

[37] Michalska A., Kisiel A., Maksymiuk K., Inzelt G., Lewenstam A., Scholz F. (2013). Screen-Printed Disposable Reference Electrodes. Handbook of Reference Electrodes.

[38] Obaje E.A., Cummins G., Schulze H., Mahmood S., Desmulliez M.P.Y., Bachmann T.T. (2016). Carbon Screen-Printed Electrodes on Ceramic Substrates for Label-Free Molecular Detection of Antibiotic Resistance. J. Interdiscip. Nanomed..

[39] Paimard G., Ghasali E., Baeza M. (2023). Screen-Printed Electrodes: Fabrication, Modification, and Biosensing Applications. Chemosensors.

[40] Kadara R.O., Jenkinson N., Banks C.E. (2009). Screen Printed Recessed Microelectrode Arrays. Sens. Actuators B Chem..

[41] Tan F., Metters J.P., Banks C.E. (2013). Electroanalytical Applications of Screen Printed Microelectrode Arrays. Sens. Actuators B Chem..

[42] Yévenes C.F.G., Wongkaew N., Ngamchana S., Surareungchai W. (2022). Exploring Interdigitated Electrode Arrays Screen-Printed on Paper Substrates for Steady-State Electrochemical Measurements. J. Electrochem. Soc..

[43] Ibáñez-Redín G., Furuta R.H.M., Wilson D., Shimizu F.M., Materon E.M., Arantes L.M.R.B., Melendez M.E., Carvalho A.L., Reis R.M., Chaur M.N. (2019). Screen-Printed Interdigitated Electrodes Modified with Nanostructured Carbon Nano-Onion Films for Detecting the Cancer Biomarker CA19-9. Mater. Sci. Eng. C.

[44] Dong H., Li C.-M., Zhang Y.-F., Cao X.-D., Gan Y. (2007). Screen-Printed Microfluidic Device for Electrochemical Immunoassay. Lab Chip.

[45] Nawaz M.A.H., Rauf S., Catanante G., Nawaz M.H., Nunes G., Marty J.L., Hayat A. (2016). One Step Assembly of Thin Films of Carbon Nanotubes on Screen Printed Interface for Electrochemical Aptasensing of Breast Cancer Biomarker. Sensors.

[46] Chan K.F., Lim H.N., Shams N., Jayabal S., Pandikumar A., Huang N.M. (2016). Fabrication of Graphene/Gold-Modified Screen-Printed Electrode for Detection of Carcinoembryonic Antigen. Mater. Sci. Eng. C.

[47] Lee S.X., Lim H.N., Ibrahim I., Jamil A., Pandikumar A., Huang N.M. (2017). Horseradish Peroxidase-Labeled Silver/Reduced Graphene Oxide Thin Film-Modified Screen-Printed Electrode for Detection of Carcinoembryonic Antigen. Biosens. Bioelectron..

[48] Chandra P., Singh J., Singh A., Srivastava A., Goyal R.N., Shim Y.B. (2013). Gold Nanoparticles and Nanocomposites in Clinical Diagnostics Using Electrochemical Methods. J. Nanoparticles.

[49] Zhang C., Zhang S., Jia Y., Li Y., Wang P., Liu Q., Xu Z., Li X., Dong Y. (2019). Sandwich-Type Electrochemical Immunosensor for Sensitive Detection of CEA Based on the Enhanced Effects of Ag NPs@CS Spaced Hemin/rGO. Biosens. Bioelectron..

[50] Nguyen V.-A., Nguyen H.L., Nguyen D.T., Do Q.P., Tran L.D. (2017). Electrosynthesized Poly(1,5-Diaminonaphthalene)/Polypyrrole Nanowires Bilayer as an Immunosensor Platform for Breast Cancer Biomarker CA 15-3. Curr. Appl. Phys..

[51] Pacheco J.G., Silva M.S.V., Freitas M., Nouws H.P.A., Delerue-Matos C. (2018). Molecularly Imprinted Electrochemical Sensor for the Point-of-Care Detection of a Breast Cancer Biomarker (CA 15-3). Sens. Actuators B Chem..

[52] Chocholova E., Bertok T., Lorencova L., Holazova A., Farkas P., Vikartovska A., Bella V., Velicova D., Kasak P., Eckstein A.A. (2018). Advanced Antifouling Zwitterionic Layer Based Impedimetric HER2 Biosensing in Human Serum: Glycoprofiling as a Novel Approach for Breast Cancer Diagnostics. Sens. Actuators B Chem..

[53] Crapnell R.D., Ferrari A.G.-M., Dempsey N.C., Banks C.E. (2022). Electroanalytical Overview: Screen-Printed Electrochemical Sensing Platforms for the Detection of Vital Cardiac, Cancer and Inflammatory Biomarkers. Sens. Diagn..

[54] Ferreira D.C., Batistuti M.R., Bachour B., Mulato M. (2021). Aptasensor Based on Screen-Printed Electrode for Breast Cancer Detection in Undiluted Human Serum. Bioelectrochemistry.

[55] Marques R.C.B., Costa-Rama E., Viswanathan S., Nouws H.P.A., Costa-García A., Delerue-Matos C., González-García M.B. (2018). Voltammetric Immunosensor for the Simultaneous Analysis of the Breast Cancer Biomarkers CA 15-3 and HER2-ECD. Sens. Actuators B Chem..

[56] Al-Khafaji Q.a.M., Harris M., Tombelli S., Laschi S., Turner A.P.F., Mascini M., Marrazza G. (2012). An Electrochemical Immunoassay for HER2 Detection. Electroanalysis.

[57] Tallapragada S.D., Layek K., Mukherjee R., Mistry K.K., Ghosh M. (2017). Development of Screen-Printed Electrode Based Immunosensor for the Detection of HER2 Antigen in Human Serum Samples. Bioelectrochemistry.

[58] Hartati Y.W., Syahruni S., Gaffar S., Wyantuti S., Yusuf M., Subroto T. (2021). An Electrochemical Aptasensor for the Detection of HER2 as a Breast Cancer Biomarker Based on Gold Nanoparticles-Aptamer Bioconjugates. Indones. J. Chem..

[59] Bezerra G., Córdula C., Campos D., Nascimento G., Oliveira N., Seabra M.A., Visani V., Lucas S., Lopes I., Santos J. (2019). Electrochemical Aptasensor for the Detection of HER2 in Human Serum to Assist in the Diagnosis of Early Stage Breast Cancer. Anal. Bioanal. Chem..

[60] Carvajal S., Fera S.N., Jones A.L., Baldo T.A., Mosa I.M., Rusling J.F., Krause C.E. (2018). Disposable Inkjet-Printed Electrochemical Platform for Detection of Clinically Relevant HER-2 Breast Cancer Biomarker. Biosens. Bioelectron..

[61] Marques R.C.B., Viswanathan S., Nouws H.P.A., Delerue-Matos C., González-García M.B. (2014). Electrochemical Immunosensor for the Analysis of the Breast Cancer Biomarker HER2 ECD. Talanta.

[62] Freitas M., Nouws H.P.A., Delerue-Matos C. (2019). Electrochemical Sensing Platforms for HER2-ECD Breast Cancer Biomarker Detection. Electroanalysis.

[63] Ravalli A., da Rocha C.G., Yamanaka H., Marrazza G. (2015). A Label-Free Electrochemical Affisensor for Cancer Marker Detection: The Case of HER2. Bioelectrochemistry.

[64] Wignarajah S., Chianella I., Tothill I.E. (2023). Development of Electrochemical Immunosensors for HER-1 and HER-2 Analysis in Serum for Breast Cancer Patients. Biosensors.

[65] Ilkhani H., Ravalli A., Marrazza G. (2016). Design of an Affibody-Based Recognition Strategy for Human Epidermal Growth Factor Receptor 2 (HER2) Detection by Electrochemical Biosensors. Chemosensors.

[66] Nemeir I.A., Mouawad L., Saab J., Hleihel W., Errachid A., Zine N. (2020). Electrochemical Impedance Spectroscopy Characterization of Label-Free Biosensors for the Detection of HER2 in Saliva. Proceedings.

[67] Oliveira A.E.F., Pereira A.C., Resende M.A.C., Ferreira L.F. (2024). Disposable Voltammetric Immunosensor for Determination and Quantification of Biomarker CA 15-3 in Biological Specimens. Analytica.

[68] Thongwattana N., Phasuksom K., Ariyasajjamongkol N., Parinyanitikul N., Sirivat A. (2024). Detection of Cancer Biomarker CA15-3 in Serums by Label-Free Immunosensor Based on Multiwall-Carbon Nanotube/Doped-Poly(2-Chloroaniline). Microchem. J..

[69] Oliveira A.E.F., Pereira A.C., Ferreira L.F. (2023). Disposable Electropolymerized Molecularly Imprinted Electrochemical Sensor for Determination of Breast Cancer Biomarker CA 15-3 in Human Serum Samples. Talanta.

[70] de Oliveira R.A.G., Materon E.M., Melendez M.E., Carvalho A.L., Faria R.C. (2017). Disposable Microfluidic Immunoarray Device for Sensitive Breast Cancer Biomarker Detection. ACS Appl. Mater. Interfaces.

[71] Gomes R.S., Moreira F.T.C., Fernandes R., Sales M.G.F. (2018). Sensing CA 15-3 in Point-of-Care by Electropolymerizing O-Phenylenediamine (oPDA) on Au-Screen Printed Electrodes. PLoS ONE.

[72] Ribeiro J.A., Pereira C.M., Silva A.F., Sales M.G.F. (2018). Disposable Electrochemical Detection of Breast Cancer Tumour Marker CA 15-3 Using Poly(Toluidine Blue) as Imprinted Polymer Receptor. Biosens. Bioelectron..

[73] Oliveira D., Barcelay Y.R., Moreira F.T.C. (2024). An Electrochemically Synthesized Molecularly Imprinted Polymer for Highly Selective Detection of Breast Cancer Biomarker CA 15-3: A Promising Point-of-Care Biosensor. RSC Adv..

[74] Khoshroo A., Mazloum-Ardakani M., Forat-Yazdi M. (2018). Enhanced Performance of Label-Free Electrochemical Immunosensor for Carbohydrate Antigen 15-3 Based on Catalytic Activity of Cobalt Sulfide/Graphene Nanocomposite. Sens. Actuators B Chem..

[75] Ma C., Li W., Kong Q., Yang H., Bian Z., Song X., Yu J., Yan M. (2015). 3D Origami Electrochemical Immunodevice for Sensitive Point-of-Care Testing Based on Dual-Signal Amplification Strategy. Biosens. Bioelectron..

[76] Shi S.-S., Li X.-J., Ma R.-N., Shang L., Zhang W., Zhao H.-Q., Jia L.-P., Wang H.-S. (2024). A Novel Dual-Signal Output Strategy for POCT of CEA Based on a Smartphone Electrochemical Aptasensing Platform. Microchim. Acta.

[77] Shamsuddin S.H., Gibson T.D., Tomlinson D.C., McPherson M.J., Jayne D.G., Millner P.A. (2021). Reagentless Affimer- and Antibody-Based Impedimetric Biosensors for CEA-Detection Using a Novel Non-Conducting Polymer. Biosens. Bioelectron..

[78] Viswanathan S., Rani C., Vijay Anand A., Ho J.A. (2009). Disposable Electrochemical Immunosensor for Carcinoembryonic Antigen Using Ferrocene Liposomes and MWCNT Screen-Printed Electrode. Biosens. Bioelectron..

[79] Pavithra M., Muruganand S., Parthiban C. (2018). Development of Novel Paper Based Electrochemical Immunosensor with Self-Made Gold Nanoparticle Ink and Quinone Derivate for Highly Sensitive Carcinoembryonic Antigen. Sens. Actuators B Chem..

[80] Wang Y., Xu H., Luo J., Liu J., Wang L., Fan Y., Yan S., Yang Y., Cai X. (2016). A Novel Label-Free Microfluidic Paper-Based Immunosensor for Highly Sensitive Electrochemical Detection of Carcinoembryonic Antigen. Biosens. Bioelectron..

[81] Feng D., Su J., He G., Xu Y., Wang C., Zheng M., Qian Q., Mi X. (2020). Electrochemical DNA Sensor for Sensitive BRCA1 Detection Based on DNA Tetrahedral-Structured Probe and Poly-Adenine Mediated Gold Nanoparticles. Biosensors.

[82] Li C., Karadeniz H., Canavar E., Erdem A. (2012). Electrochemical Sensing of Label Free DNA Hybridization Related to Breast Cancer 1 Gene at Disposable Sensor Platforms Modified with Single Walled Carbon Nanotubes. Electrochim. Acta.

[83] Raucci A., Cimmino W., Grosso S.P., Normanno N., Giordano A., Cinti S. (2024). Paper-Based Screen-Printed Electrode to Detect miRNA-652 Associated to Triple-Negative Breast Cancer. Electrochim. Acta.

[84] Pimalai D., Putnin T., Waiwinya W., Chotsuwan C., Aroonyadet N., Japrung D. (2021). Development of Electrochemical Biosensors for Simultaneous Multiplex Detection of microRNA for Breast Cancer Screening. Microchim. Acta.

[85] Rebelo T.S.C.R., Pereira C.M., Sales M.G.F., Noronha J.P., Costa-Rodrigues J., Silva F., Fernandes M.H. (2014). Sarcosine Oxidase Composite Screen-Printed Electrode for Sarcosine Determination in Biological Samples. Anal. Chim. Acta.

[86] Hu F., Liu T., Pang J., Chu Z., Jin W. (2020). Facile Preparation of Porous Co3O4 Nanocubes for Directly Screen-Printing an Ultrasensitive Glutamate Biosensor Microchip. Sens. Actuators B Chem..

[87] Li M., Li D.-W., Xiu G., Long Y.-T. (2017). Applications of Screen-Printed Electrodes in Current Environmental Analysis. Curr. Opin. Electrochem..

[88] Mincu N.-B., Lazar V., Stan D., Mihailescu C.M., Iosub R., Mateescu A.L. (2020). Screen-Printed Electrodes (SPE) for In Vitro Diagnostic Purpose. Diagnostics.

[89] Rebelo T.S.C.R., Costa R., Brandão A.T.S.C., Silva A.F., Sales M.G.F., Pereira C.M. (2019). Molecularly Imprinted Polymer SPE Sensor for Analysis of CA-125 on Serum. Anal. Chim. Acta.

[90] Mpupa A., Selahle S.K., Mizaikoff B., Nomngongo P.N. (2021). Recent Advances in Solid-Phase Extraction (SPE) Based on Molecularly Imprinted Polymers (MIPs) for Analysis of Hormones. Chemosensors.

